# Brightening and Dimming Aftereffects at Low and High Luminance

**DOI:** 10.3390/vision2020024

**Published:** 2018-06-13

**Authors:** Omar Hassan, Mark A. Georgeson, Stephen T. Hammett

**Affiliations:** 1 Department of Psychology, Royal Holloway, University of London, Egham TW20 0EX, UK; 2 School of Life & Health Sciences, Aston University, Birmingham B4 7ET, UK

**Keywords:** adaptation, luminance, ramp, flicker, temporal frequency, motion

## Abstract

Adaptation to a spatially uniform field that increases or decreases in luminance over time yields a “ramp aftereffect”, whereby a steady, uniform luminance appears to dim or brighten, and an appropriate non-uniform test field appears to move. We measured the duration of this aftereffect of adaptation to ascending and descending luminance for a wide range of temporal frequencies and luminance amplitudes. Three types of luminance ramp profiles were used: linear, logarithmic, and exponential. The duration of the motion aftereffect increased as amplitude increased, regardless of the frequency, slope, or ramp profile of the adapting pattern. At low luminance, this result held for ascending luminance adaptation, but the duration of the aftereffect was significantly reduced for descending luminance adaptation. This reduction in the duration of the aftereffect at low luminance is consistent with differential recruitment of temporally tuned cells of the ON and OFF pathways, but the relative independence of the effect from temporal frequency is not.

## 1. Introduction

It is well known that the visual system adjusts its sensitivity to the prevailing level of illumination. Adaptation does not occur instantly; moving from a dark environment to a light one or vice versa requires a short period of time before the visual system adjusts to seeing at the new level of illumination. These processes are known as light and dark adaptation, respectively. In 1967, Anstis [1] reported a new effect in which the visual system adapts not to the mean intensity of illumination but to changing illumination. The neural substrate of these brightening and dimming aftereffects has yet to be determined.

**Figure 1:**
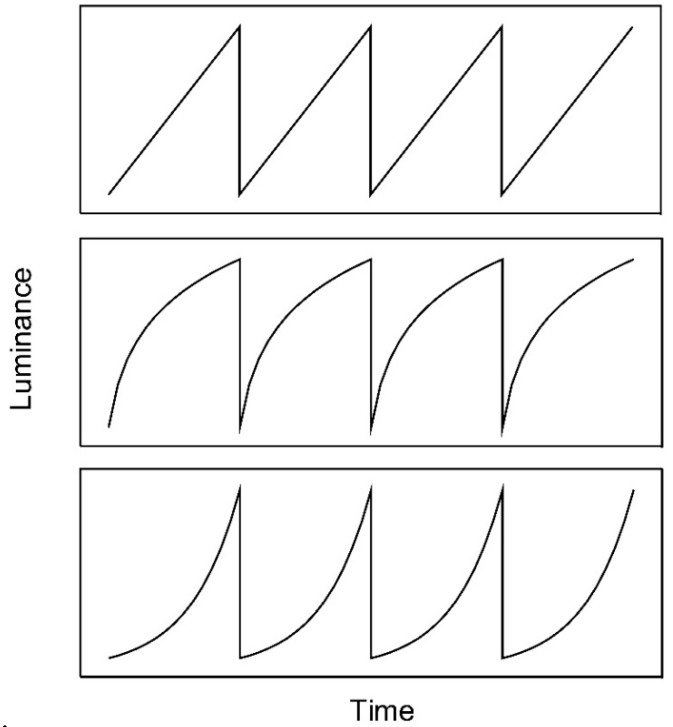
Linear (**upper panel**), logarithmic (**middle panel**), and exponential (**lower panel**) luminance ramps. Linear and exponential luminance ramps were used by Anstis [1] and Arnold and Anstis [4], respectively.

Anstis measured the effect of adapting to stimuli whose luminance increased or decreased over time as a sawtooth function (Figure 1). He found that after adapting to a spatially uniform field of light that gradually brightened, a subsequently viewed uniform test field of constant luminance appeared to dim over time. Conversely, adaptation to a gradually dimming pattern yielded a percept of a gradual brightening in a constant test stimulus. This phenomenon is termed the ramp aftereffect. Anstis reported informally that the temporal frequency and amplitude of luminance modulation were not critical to the effect, but that a temporal frequency of 1 Hz and a high amplitude (40 dB) were near optimum for eliciting the longest aftereffect duration. This was the only combination of frequency and amplitude tested by Anstis.

Anstis also reported that, following adaptation to a luminance ramp, a static test field that contained a spatial luminance gradient appeared to move [2]. Viewing a spatially uniform adapting field, the luminance of which was modulated by an ascending sawtooth ramp, followed by a test field consisting of a spatial luminance gradient darkest on the left, rightward motion was perceived along with apparent dimming. Conversely, viewing a test field consisting of a spatial luminance gradient darkest on the right yielded apparent dimming and leftward motion. However, without a spatial luminance gradient present in the test field, no motion was perceived, only dimming. Anstis concluded that this motion aftereffect was an artefact due to the presence of a post-adaptation spatial luminance gradient. The ascending luminance ramp adapts the visual system to a continually brightening scene. As such, the parts of the visual system that code for increasing luminance reduce their baseline activity, tilting the balance in favour of descending luminance signals. Thus, a post-adaptation spatially uniform test field appears to be dimming. Krauskopf and Zaidi’s [3] finding that sawtooth adaptation also yields polarity-specific reductions in contrast sensitivity is consistent with such a scheme. With a spatial luminance gradient presented after adaptation, the combination of real spatial gradient and illusory temporal gradient is sufficient to generate a motion signal.

Arnold and Anstis [4] further investigated the ramp aftereffect using a nulling method rather than the duration of the aftereffect. Adapting stimuli comprised a spatially uniform field the luminance of which was modulated by a sawtooth waveform. After adaptation, luminance ramps of the same polarity as the adapting pattern were presented in order to cancel the illusory appearance of an opposite ramp. The amplitude of these nulling ramps was varied until test fields appeared to be neither dimming nor brightening. Using this method, they found that the aftereffect was proportional to the amplitude of the adapting waveform and not its gradient or temporal frequency. However, since the gradient of the exponential luminance ramp (Figure 1) used by Arnold and Anstis varied systematically over time, it is not trivial to determine whether gradient or contrast is the key factor. 

The question of where in the visual system these aftereffects arise is yet to be resolved, but a number of studies have provided (sometimes paradoxical) clues. The ramp aftereffect does not transfer interocularly [1,5] which implies a sub-cortical or very early cortical (V1) locus of the underlying mechanism, as it is commonly believed that there are no cells that receive monocular input in any visual cortical areas other than V1 [6,7,8]. Although contrast adaptation of cells in the visual cortex is well established, contrast adaptation in pre-cortical cells remained elusive until relatively recently. However, it is now clear that contrast adaptation is widely found in the retinae of non-mammalian [9,10] and mammalian species [11], including primates [12].

It is tempting to conclude that ramp aftereffects have their origin at an early (subcortical) location within the visual system given the lack of interocular transfer and the physiological evidence for subcortical contrast adaptation. Indeed, a number of studies suggested that the aftereffect is mediated by magnocellular ON and OFF responses [4,5]. However, Anstis and Harris’s finding that the receptive field size associated with these aftereffects is very large [5] (over 20 times the diameter of the cells underlying acuity) is not compatible with any known subcortical or early cortical neurons. Their findings indicated that receptive field size radius (*r*) underlying the aftereffect increased with eccentricity (*e*) such that *r = 6.17e − 0.51*, whereas primate magnocellular receptive field radii are estimated to increase such that *r = 0.07e + 0.058* [13]. At 10 degrees eccentricity, the area of the receptive field underlying the ramp aftereffect is many times greater than that of primate magnocellular receptive fields, more akin to receptive field sizes found in much higher cortical levels such as the middle temporal area (MT). Thus, two key pieces of evidence point to very different locations for the origin of these aftereffects—the lack of interocular transfer suggests that the neural substrate of these effects is sub-cortical, whereas the very large receptive field size points to a much later cortical location. 

In order to characterise more fully the attributes of the phenomenon, we have measured the effect of mean luminance level (known to influence ON and OFF channels differentially [14]) and temporal frequency (known to influence magnocellular sensitivity [15]) on the ramp aftereffect. We measured the aftereffect duration (appearance of motion on a test ramp) for a range of adapting amplitudes and gradients (Experiment 1) and for linear, logarithmic, and exponential ramps (Experiment 2).

## 2. Materials and Methods

### 2.1. Subjects

Five (two male and three female) subjects, aged between 20 and 27 years, participated in this experiment. One of the subjects (OH) was an author, while the other four were naïve to the purpose of the experiment. All subjects had normal or corrected-to-normal acuity. Informed consent of subjects was obtained prior to experimentation. The work was carried out in accordance with the Code of Ethics of the World Medical Association (Declaration of Helsinki).

### 2.2. Apparatus and Stimuli

Stimuli were generated using the Psychophysics Toolbox extensions [16,17,18] for MATLAB 7.11 (MathWorks, Cambridge, UK) and displayed on an EIZO 6600-M (Hakusan, Ishikawa, Japan) monochrome monitor at a frame rate of 60 Hz. The monitor was gamma corrected using the CRS OptiCal photometric system (Cambridge Research Systems, Rochester, UK). The display subtended 47° × 34° at a viewing distance of 40 cm. Mean luminance was 24.75 cd m^−2^ for the high luminance conditions and 2.475 cd m^−2^ in the low luminance conditions. In the low luminance conditions, 1 log unit neutral density filters (NDF) (Thorlabs Inc., Newton, NJ, USA) were inserted into drop-cell trial frames (Skeoch, Sussex, UK) that were worn by subjects in all conditions.

**Figure 2:**
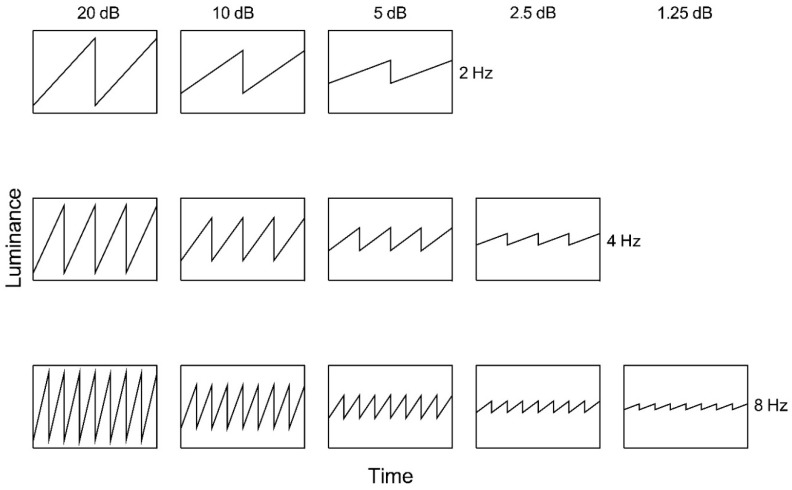
Ascending sawtooth luminance waveforms as a function of time. Nominal amplitude is indicated above each column and temporal frequency to the right of each row.

Adapting stimuli (Figure 2) comprised spatially uniform fields subtending 47° × 34° (horizontal × vertical). Luminance was modulated using ascending and descending sawtooth waveforms at 2, 4, or 8 Hz. The amplitude of luminance modulation ranged across 1.25, 2.5, 5, 10, and 20 decibels (dB), where

$${\mathrm{dB}=20\log}_{10}\left(\frac{\mathrm{Lmax}}{\mathrm{Lmin}}\right)$$

where Lmax and Lmin are maximum and minimum luminance, respectively. The amplitude ranges correspond to Michelson contrasts (m) of 0.072, 0.143, 0.28, 0.519, and 0.818, respectively, where

$$m=\frac{\mathrm{Lmax}-\mathrm{Lmin}}{\mathrm{Lmax}+\mathrm{Lmin}}$$

Adapting amplitudes of 2.5 dB and 1.25 dB were approaching the threshold for 2 Hz stimuli and were thus not used, and similarly, adapting amplitudes of 1.25 dB were not used at 4 Hz. All test stimuli consisted of a 0.023 c/deg horizontally oriented half-cosine edge, subtending 25° × 22° (horizontal × vertical) with a nominal Michelson contrast of 1 (actual 0.99). The remainder of the screen was presented at mean luminance.

### 2.3. Procedure

Before beginning the experiment, subjects were dark adapted for at least 5 min. A trial consisted of an adaptation interval of 16 s followed by a test interval. In different conditions, the stimuli were presented at high and low luminance for ascending and descending linear luminance ramps. Each trial block consisted of a single temporal frequency and mean luminance. Within each block, the amplitude of luminance modulation was randomised from trial to trial. The frequency and luminance tested were randomised from block to block. Subjects were required to fixate on the centre of the screen during the adaptation and test intervals and to indicate when the perception of motion had ceased during the test interval by pressing a mouse button. The test edge spatial phase was switched between 0° and 180° (darkest at the top or darkest at the bottom) across trials. A blank screen of mean luminance was presented for at least 30 s between each trial. The mean of five estimates was taken as the duration of the aftereffect for each condition. Ten practice trials were presented at the beginning of each trial block. The experiments were conducted binocularly in a semidarkened room using a chin and head rest.

The experimental details for Experiment 2 were identical to those of Experiment 1, except that only the high luminance condition was included and the profile of adaptation luminance was modulated using ascending and descending logarithmic and exponential waveforms as opposed to linear waveforms (Figure 1). As before, each trial block consisted of a single luminance ramp profile.

## 3. Experiment 1: The Effect of Adaptation Frequency, Amplitude, and Luminance upon the Ramp Aftereffect

An understanding of which parameters of the adapting stimulus determine the ramp aftereffect may provide important clues to the underlying physiological substrate of the phenomenon which, in turn, may yield insight into a fundamental aspect of the visual system’s ability to adapt to prevailing dynamic conditions. For instance, Anstis [1] speculated that the effect may be mediated by “ON” and “OFF” retinal cells. Arnold and Anstis [4] suggested that ascending luminance ramps selectively adapt the ON-channels, whereas descending ramps selectively adapt the OFF-channels. In line with this argument, adapting to ascending ramps reduced detectability of luminance increments, while falling ramps reduced sensitivity to decrements [19]. This proposition can be further tested by comparing the aftereffect duration induced by ascending and descending luminance ramps at high and low luminance. Since OFF cell responses are known to be reduced at low luminance [14,20,21], we measured the aftereffect duration at both high and low luminance using a graded test field to induce illusory motion.

### Results

**Figure 3:**
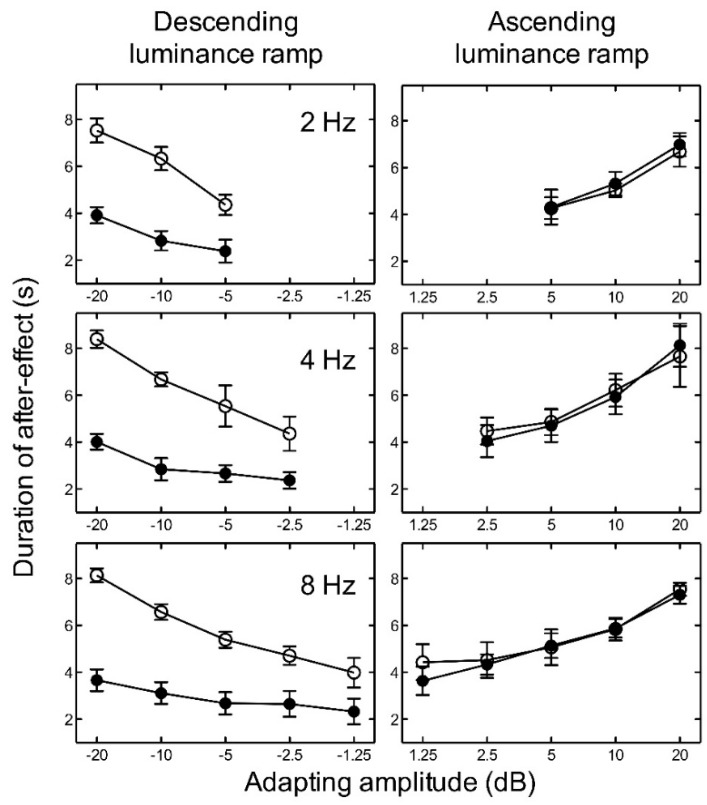
Experiment 1. The average duration of the aftereffect is plotted as a function of adapting luminance amplitude for high luminance (open circles) and low luminance (closed circles) with adapting frequencies of 2, 4, and 8 Hz. Luminance amplitudes greater than zero represent ascending ramps and negative values represent descending ramps. Error bars represent ±1 SEM across the five observers.

For ascending luminance ramps, the results indicate that as the amplitude of the adapting ramp increased, the duration of the aftereffect also increased for all temporal frequencies tested (Figure 3). Since the number of amplitude levels tested varied with temporal frequency, we conducted several two-way ANOVAs. In order to investigate the effect of luminance level and frequency, we averaged across the adapting amplitudes for each participant to give one duration value for low luminance and one for high luminance at each frequency. A two-way repeated measures ANOVA revealed no significant main effect of luminance level, *F*(1, 8) = 0.00, *p* = 0.931; no significant main effect of frequency, *F*(2, 8) = 0.69, *p* = 0.527; and no significant interaction between luminance and frequency, *F*(2, 8) = 0.31, *p* = 0.736, on the duration of the aftereffect. For descending luminance ramps, the aftereffect duration again increased with adapting amplitude (plotted as negative values in Figure 3), but also depended very markedly on luminance level. At low luminance, the aftereffect duration was more than a factor of two shorter than at high luminance. A two-way repeated measures ANOVA revealed a significant main effect of luminance, *F*(1, 8) = 41.74, *p* < 0.005; but no significant main effect of frequency, *F*(2, 8) = 0.62, *p* = 0.561; and no significant interaction between luminance and frequency, *F*(2, 8) = 0.53, *p* = 0.604, on the duration of the aftereffect.

For each temporal frequency at high luminance, two-way repeated measures ANOVAs revealed no significant main effect of adapting luminance ramp direction (ascending or descending), a significant main effect of ramp amplitude, and no significant interaction between ramp direction and ramp amplitude on the duration of the aftereffect (Table 1).

For each adapting amplitude, the duration of the aftereffect was fairly constant across the three adapting temporal frequencies. The ramp aftereffect appears to be closely related to adapting amplitude and relatively independent of adapting temporal frequency. The maximum duration of the aftereffect was consistently found at peak amplitude, irrespective of frequency, suggesting that the aftereffect duration is determined by ramp amplitude rather than temporal frequency or temporal gradient.

However, since temporal gradient is proportional to the product of temporal frequency and amplitude, we evaluated the relative contribution of gradient and amplitude quantitatively. Two linear regression analyses were run to examine the relationships between duration of the aftereffect and (a) ramp amplitude and (b) ramp gradient. For the amplitude model, *R*^2^ = 0.481, *F*(1, 118) = 109.22, *p* < 0.001. For the gradient model, *R*^2^ = 0.332, *F*(1, 118) = 58.53, *p* < 0.001. Thus, both models could explain a significant proportion of the variance in duration of the aftereffect. The correlation between the two models was significant (r = 0.732, *p* < 0.001). The predictive utility of the two models was therefore compared using Hotelling’s t-test for nonindependent correlations. The results indicate that the amplitude model accounted for significantly more variance than the gradient model, t(117) = 2.40, *p* < 0.050.

## 4. Experiment 2: The Effect of Linear, Logarithmic, and Exponential Luminance Ramps upon the Ramp Aftereffect

Whilst Arnold and Anstis [4] suggested that the aftereffect was strongly driven by amplitude, they used exponential adapting ramps in which the gradient varied with time, whereas Anstis [1] used linear ramps (Figure 1). Such differences in ramp profile might influence which parameters of the visual scene determine the strength of the ramp aftereffect. Furthermore, a relatively limited range of parameters has been previously studied. For instance, Arnold and Anstis measured the effect between 0.5 and 4 Hz for amplitudes ranging from 5 to 20 dB using logarithmic ramps, whereas Anstis used a linear ramp of 1 Hz and 40 dB (Figure 1). Since one possible neural substrate of the aftereffect is neurones tuned to specific temporal gradients, the question arises as to whether changes in gradient profile (rather than magnitude) might influence the aftereffect. If specific gradient tuned mechanisms underlie the aftereffect, then changes in gradient profile may well reveal changes in the aftereffect under conditions where gradient magnitude is held constant. In order to evaluate the effect of these ramp characteristics on the duration of the aftereffect, we therefore measured the effect of luminance ramp adaptation for a broader range of frequencies (2–8 Hz) and luminance amplitudes (1.25–20 dB) for both ascending and descending, linear, logarithmic, and exponential luminance ramp profiles at high (24.75 cd m^−2^) luminance.

### Results

**Figure 4:**
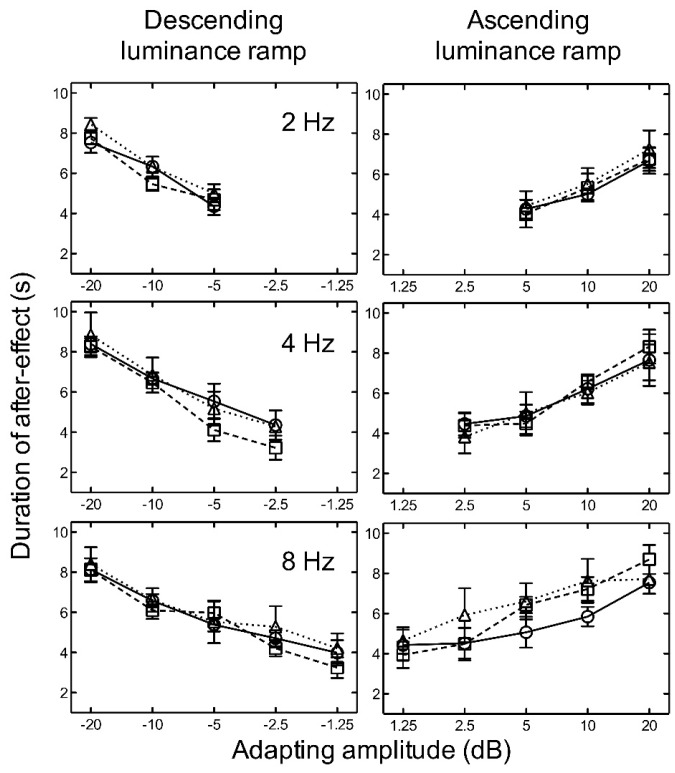
Experiment 2. The duration of the aftereffect is plotted as a function of adapting luminance amplitude for linear (circles/solid line) (from Experiment 1), logarithmic (squares/broken line), and exponential (triangles/dotted line) luminance ramps at 2, 4, and 8 Hz. Luminance amplitudes greater than zero represent ascending ramps and negative values represent descending ramps. Symbols represent the mean of five subjects and error bars represent ±1 SEM.

For both ascending and descending linear, logarithmic, and exponential luminance ramp adaptation, the results indicate that as amplitude increased, the aftereffect duration increased for all temporal frequencies tested (Figure 4).

There was no significant difference between the durations of the aftereffect after adaptation to linear, logarithmic, or exponential ramps (Figure 4). Two-way repeated measures ANOVAs revealed no significant main effects of luminance ramp profile for ascending, *F*(2, 16) = 0.24, *p* = 0.786, nor descending, *F*(2, 16) = 2.42, *p* = 0.150, stimuli. No significant main effects of frequency (ascending: *F*(2, 16) = 2.56, *p* = 0.138) (descending: *F*(2, 16) = 1.75, *p* = 0.233), and no significant interactions between luminance ramp profile and frequency (ascending: *F*(4, 16) = 0.91, *p* = 0.479), (descending: *F*(4, 16) = 0.23, *p* = 0.915), were found. 

## 5. Discussion

The results of both experiments indicate that the ramp aftereffect increases as the amplitude of the ramp increases. One possibility suggested by a number of studies [1,4] is that the aftereffect is mediated by adapting the ON and OFF pathways in the visual system that are selectively tuned for increments and decrements in luminance. ON retinal ganglion cells are stimulated by local increments in illumination, whereas OFF ganglion cells respond to local decrements in illumination [20,21,22,23]. Thus, the ramp aftereffect might be due to selective adaptation of the ON or OFF cells by ascending or descending luminance ramps. We find that whilst adaptation to ascending luminance ramps yielded a similar pattern of results at high and low luminance, adaptation to descending ramps yielded a significantly shorter aftereffect (by about a factor of two) at low luminance. How could this difference be explained? The response properties of cortical ON and OFF cells are significantly altered by absolute luminance level. At low luminance, OFF cell responses are reduced or absent, while ON responses are unaffected [14,20,21]. We find a significant reduction in the ramp aftereffect under just such conditions—at low luminance and exclusively for descending (OFF) luminance ramps.

The significant reduction in the duration of the aftereffect for descending ramps at low luminance is consistent with selective adaptation of ON and OFF pathways. However, since ON and OFF pathways are found from retina to cortex [21,23] this does not help to identify the location of the effect. Moreover, the results also indicate that the duration of the aftereffect is well characterised by the amplitude of the adapting ramp—the same amplitude yields very similar aftereffects regardless of the frequency of adaptation. This is perhaps surprising since the sensitivity of both magnocellular and parvocellular cells rises monotonically over the range of frequencies tested here [15]. Thus, if the ramp aftereffects were due to adaptation of selective ON and OFF response magnocellular cells, as has previously been suggested [4] (or for that matter, parvocellular cells), then we might expect the effect to be tuned for temporal frequency. This was not the case. Rather, regardless of adaptation frequency, the maximum duration of the aftereffect was found at the maximum amplitude. 

Our finding that mean luminance level differentially modulates the effect of descending ramp adaptation strongly suggests that it is mediated by the OFF pathways, but the lack of any temporal frequency tuning does not seem consistent with the effect originating in the response of magnocellular cells. One other potential substrate for the effect may be specialised luminance gradient tuned cells. Anstis [24] concluded that since ramp aftereffects can be perceived as motion aftereffects, “motion detectors include a filter to detect gradual change of luminance, dI/dT” (p. 65), and several other researchers [25,26] have suggested that the ramp aftereffect is consistent with a gradient-based model of motion encoding (e.g., [27]). This proposal is predicated upon the assumption that the neural mechanism which ordinarily detects motion also mediates the ramp aftereffect. The results of Experiment 1, however, show that it is amplitude rather than gradient that determines the duration of illusory motion, and the results of Experiment 2 reveal that the gradient profile does not influence the aftereffect. 

Our results demonstrate that the visual system processes brightness within a system that explicitly represents, and adapts to, the amplitude of luminance change over time and is most adapted by large amplitudes. Neither the temporal frequency nor local gradient of these changes appears critical to the system’s response over the range tested. Many aspects of the aftereffect point to an early neural substrate (e.g., lack of interocular transfer), but others (e.g., receptive field size, weak temporal tuning) suggest a much later origin. Intriguingly, recent evidence [19] suggests that the neural mechanism responsible for changes in contrast sensitivity [3] after ramp adaptation is independent of the mechanism responsible for the ramp aftereffects. These paradoxical findings raise the possibility that perhaps different locations in the visual system may be responsible for different phenomenological aspects of the aftereffect. Regardless, our results implicate the retino-cortical system of ON and OFF pathways in the mediation of the ramp aftereffect, but its precise location or locations remain elusive.

## Figures and Tables

**Table 1:** Experiment 1. Two-way repeated measures ANOVA for adapting luminance ramp direction and ramp amplitude on the duration of the aftereffect at each temporal frequency at high luminance.

Temporal Frequency					
2 Hz	Source	*df*	Mean square	F	*p*
	Luminance ramp direction	1	4.209	1.41	0.300
	Luminance ramp amplitude	2	19.460	22.89	0.000 **
	Interaction (direction x amplitude)	2	0.953	0.89	0.444
	Error	8	0.850		
4 Hz	Source	*df*	Mean square	F	*p*
	Luminance ramp direction	1	1.951	0.36	0.580
	Luminance ramp amplitude	3	24.815	5.82	0.010 *
	Interaction (direction x amplitude)	3	0.373	0.67	0.585
	Error	12	0.556		
8 Hz	Source	*df*	Mean square	F	*p*
	Luminance ramp direction	1	0.962	0.25	0.642
	Luminance ramp amplitude	4	21.055	21.60	0.000 **
	Interaction (direction x amplitude)	4	0.521	2.56	0.078
	Error	16	0.202		

* The effect is significant at the 0.01 level. ** The effect is significant at the 0.001 level.
